# Subtyping Hyperchloremia among Hospitalized Patients by Machine Learning Consensus Clustering

**DOI:** 10.3390/medicina57090903

**Published:** 2021-08-30

**Authors:** Charat Thongprayoon, Voravech Nissaisorakarn, Pattharawin Pattharanitima, Michael A. Mao, Andrea G. Kattah, Mira T. Keddis, Carissa Y. Dumancas, Saraschandra Vallabhajosyula, Tananchai Petnak, Stephen B. Erickson, John J. Dillon, Vesna D. Garovic, Kianoush B. Kashani, Wisit Cheungpasitporn

**Affiliations:** 1 Division of Nephrology and Hypertension, Department of Medicine, Mayo Clinic, Rochester, MN 55905, USA; charat.thongprayoon@gmail.com (C.T.); kattah.andrea@mayo.edu (A.G.K.); dumancas.carissa@mayo.edu (C.Y.D.); erickson.stephen@mayo.edu (S.B.E.); dillon.John@mayo.edu (J.J.D.); garovic.Vesna@mayo.edu (V.D.G.); kashani.Kianoush@mayo.edu (K.B.K.); 2 Department of Internal Medicine, MetroWest Medical Center, Tufts University School of Medicine, Boston, MA 01702, USA; voravech.niss@gmail.com; 3 Department of Internal Medicine, Faculty of Medicine, Thammasat University, Pathum Thani 12120, Thailand; pattharawin@hotmail.com; 4 Division of Nephrology and Hypertension, Department of Medicine, Mayo Clinic, Jacksonville, FL 32224, USA; mao.michael@mayo.edu; 5 Division of Nephrology and Hypertension, Department of Medicine, Mayo Clinic, Phoenix, AZ 85054, USA; keddis.Mira@mayo.edu; 6 Section of Cardiovascular Medicine, Department of Medicine, Wake Forest University School of Medicine, Winston-Salem, NC 27101, USA; svallabh@wakehealth.edu; 7 Division of Pulmonary and Critical Care Medicine, Department of Medicine, Mayo Clinic, Rochester, MN 55905, USA; petnak@yahoo.com

**Keywords:** hyperchloremia, chloride, artificial intelligence, clustering, mortality, machine learning, hospitalization

## Abstract

*Background and Objectives*: Despite the association between hyperchloremia and adverse outcomes, mortality risks among patients with hyperchloremia have not consistently been observed among all studies with different patient populations with hyperchloremia. The objective of this study was to characterize hyperchloremic patients at hospital admission into clusters using an unsupervised machine learning approach and to evaluate the mortality risk among these distinct clusters. *Materials and Methods*: We performed consensus cluster analysis based on demographic information, principal diagnoses, comorbidities, and laboratory data among 11,394 hospitalized adult patients with admission serum chloride of >108 mEq/L. We calculated the standardized mean difference of each variable to identify each cluster’s key features. We assessed the association of each hyperchloremia cluster with hospital and one-year mortality. *Results*: There were three distinct clusters of patients with admission hyperchloremia: 3237 (28%), 4059 (36%), and 4098 (36%) patients in clusters 1 through 3, respectively. Cluster 1 was characterized by higher serum chloride but lower serum sodium, bicarbonate, hemoglobin, and albumin. Cluster 2 was characterized by younger age, lower comorbidity score, lower serum chloride, and higher estimated glomerular filtration (eGFR), hemoglobin, and albumin. Cluster 3 was characterized by older age, higher comorbidity score, higher serum sodium, potassium, and lower eGFR. Compared with cluster 2, odds ratios for hospital mortality were 3.60 (95% CI 2.33–5.56) for cluster 1, and 4.83 (95% CI 3.21–7.28) for cluster 3, whereas hazard ratios for one-year mortality were 4.49 (95% CI 3.53–5.70) for cluster 1 and 6.96 (95% CI 5.56–8.72) for cluster 3. *Conclusions*: Our cluster analysis identified three clinically distinct phenotypes with differing mortality risks in hospitalized patients with admission hyperchloremia.

## 1. Introduction

Chloride constitutes 70% of the anions of the human body [1]. While chloride is distributed among various fluid compartments, it is abundant in the extracellular fluid, including plasma and interstitial fluid [2]. Chloride plays a vital role in sustaining osmolarity, electro-neutrality, and acid–base balance [2,3]. Hyperchloremia, defined as plasma chloride >108 mEq/L [3,4], is a common electrolyte disturbance with a prevalence of 15% on hospital admission [3]. This condition has increasingly been recognized as studies have demonstrated associations of hyperchloremia with decreased renal blood flow [5,6], increased interstitial edema [7], and poor clinical outcomes [8,9,10].

Patients with hyperchloremia are heterogeneous [11]. There are a different group of clinical conditions that can cause hyperchloremia, including excessive chloride administration (such as 0.9% NaCl solution), water depletion over chloride loss (such as osmotic diuresis), and normal anion gap metabolic acidosis (such as diarrhea and renal tubular acidosis (RTA)) [12]. While hyperchloremia is associated with poor clinical outcomes [8,9,10], the association between hyperchloremia and mortality has not been consistently observed among all studies with different patient populations with hyperchloremia [2,9,13,14,15,16,17,18,19,20,21,22]. Thus, identifying distinct phenotypes of hyperchloremia may help identify subgroups of hyperchloremic patients who carry an increased risk of mortality. 

Machine learning (ML) algorithms have recently been introduced to healthcare and are promising for identifying patterns that might not be discoverable using traditional statistical practices [23,24,25,26,27,28,29,30]. Among heterogeneous groups of patients, unsupervised ML clustering algorithms can be utilized to identify similarities in the patients’ characteristics, group similar data points together, and provide insight into underlying patterns of different patient groups [31,32,33]. Thus, in this study, we utilized an unsupervised ML clustering approach to identify the clusters of hospitalized patients with admission hyperchloremia and to evaluate mortality risk among these distinct clusters.

## 2. Materials and Methods

### 2.1. Patient Population

The Mayo Clinic Institutional Review Board approved this study. Adult patients (age ≥18 years) admitted at Mayo Clinic Hospital, Rochester, Minnesota, USA, from January 2011 to December 2013 were reviewed. The inclusion criterion was the presence of hyperchloremia, defined as serum chloride ≥108 mEq/L, at hospital admission. We excluded patients with (1) lack of serum chloride measurement within 24 h of hospital admission and (2) no authorization for research use.

### 2.2. Data Collection

Pertinent demographic information, principal diagnoses, comorbidities, and laboratory data were abstracted from our hospital’s electronic database using a previously validated method [3,4]. Data for cluster analysis were restricted to within 24 h of hospital admission because the study aimed to cluster hyperchloremic patients based on admission information. The first laboratory value in each 24-hour time frame was used when there were multiple available values. Variables with over 20% missing data were excluded. If a variable had missing data less than 20%, missing data were imputed using a random forest multiple imputation approach before entering cluster analysis.

### 2.3. Clustering Analysis

An unsupervised ML with a consensus clustering approach was applied to develop clinical phenotypes of hyperchloremic patients [34]. A pre-specified subsampling parameter of 80% with 100 iterations and the number of potential clusters (*k*) ranging from 2 to 10 were used to avoid producing an excessive number of clusters that would not be clinically useful. The optimal number of clusters was determined by examining the consensus matrix (CM) heat map, cumulative distribution function (CDF), cluster-consensus plots with the within-cluster consensus scores, and the proportion of ambiguously clustered pairs (PAC). The within-cluster consensus score, ranging between 0 and 1, was defined as the average consensus value for all pairs of individuals belonging to the same cluster [35]. A value closer to one indicated better cluster stability. PAC, ranging between 0 and 1, was calculated as the proportion of all sample pairs with consensus values falling within the predetermined boundaries [36]. A value closer to zero indicated better cluster stability [36]. The PAC was calculated using two criteria: 1) the strict criterion, consisting of a predetermined boundary of (0, 1), where a pair of individuals who had a consensus value >0 or <1 was considered ambiguously clustered, and 2) the relaxed criterion, consisting of a predetermined boundary of (0.1, 0.9), where a pair of individuals who had consensus value >0.1 or <0.9 was considered ambiguously clustered [36]. The detailed consensus cluster algorithms used in this study for reproducibility are provided in Online Supplement.

### 2.4. Statistical Analysis

After hyperchloremia was clustered, subsequent analyses focused on characterizing differences in characteristics and outcomes among the identified clusters. Clinical characteristics were compared among the clusters using analysis of variance (ANOVA) for continuous variables and the chi-squared test for categorical variables. To explore the key features of each cluster, the standardized mean differences of clinically relevant and readily available characteristics were calculated between each cluster and the overall population. Variables with an absolute standardized mean difference of >0.3 were considered as key features for each cluster. Hospital mortality and one-year mortality were compared among the clusters. The odds ratios for hospital mortality of clusters were obtained using logistic regression. The hazard ratio for one-year mortality in each cluster was calculated using Cox proportional hazard regression. We used cluster 2 as the reference group, as this cluster had the lowest mortality risk. Between-group differences in clinical characteristics were not adjusted because these variables were used to cluster hyperchloremia patients through an unsupervised consensus clustering approach. All analyses were performed using R, version 4.0.3 (RStudio, Inc., Boston, MA, USA; 2005. Available from: http://www.rstudio.com/, accessed on 21 July 2021), with ConsensusClusterPlus package (version 1.46.0) for consensus clustering analysis, and the missForest package for missing data imputation.

## 3. Results

Of 76,696 adult patients admitted to the hospital from 2011 to 2013, 11,394 (15%) were hyperchloremic (serum chloride > 108 mEq/L) at hospital admission. The mean age (SD) was 61 (18) years, and 50% were male. The mean admission serum chloride was 110 (3) mEq/L.

**Figure 1:**
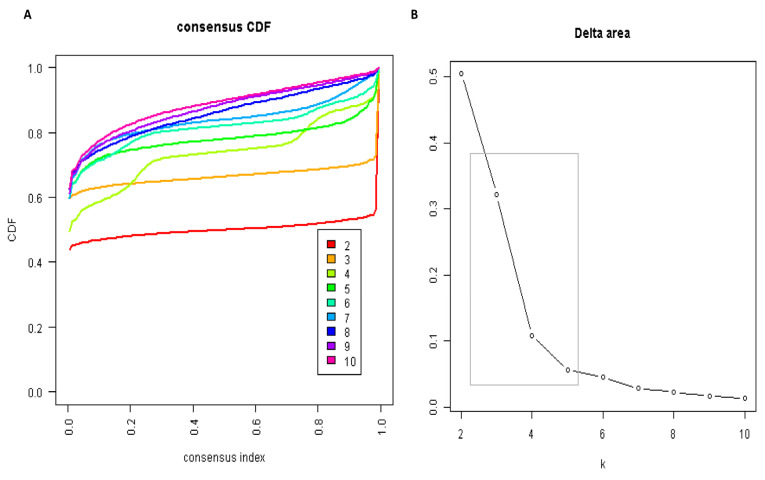
(**A**) CDF plot displaying consensus distributions for each k; (**B**) Delta area plot reflecting the relative changes in the area under the CDF curve.

**Figure 2:**
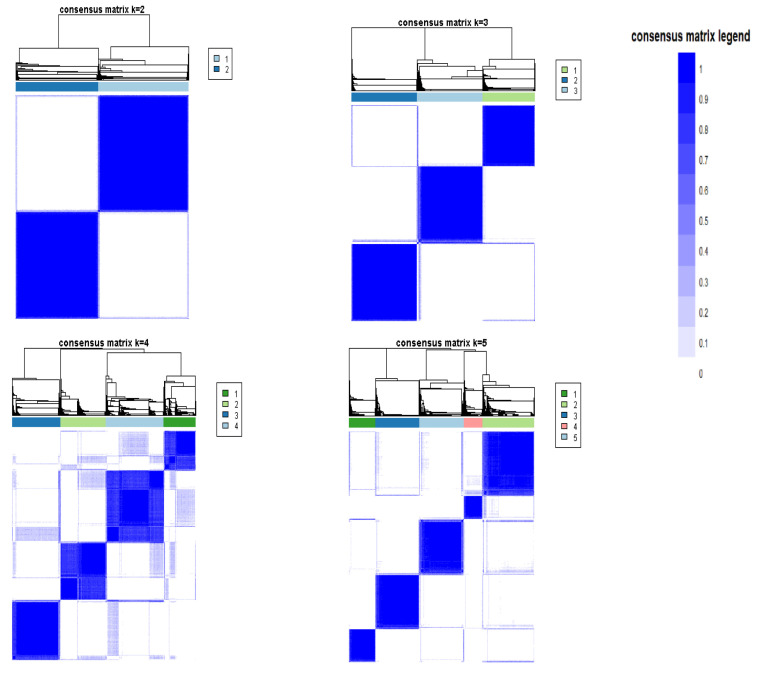
Consensus matrix heat map depicting consensus values on a white to blue color scale of each cluster.

**Figure 3:**
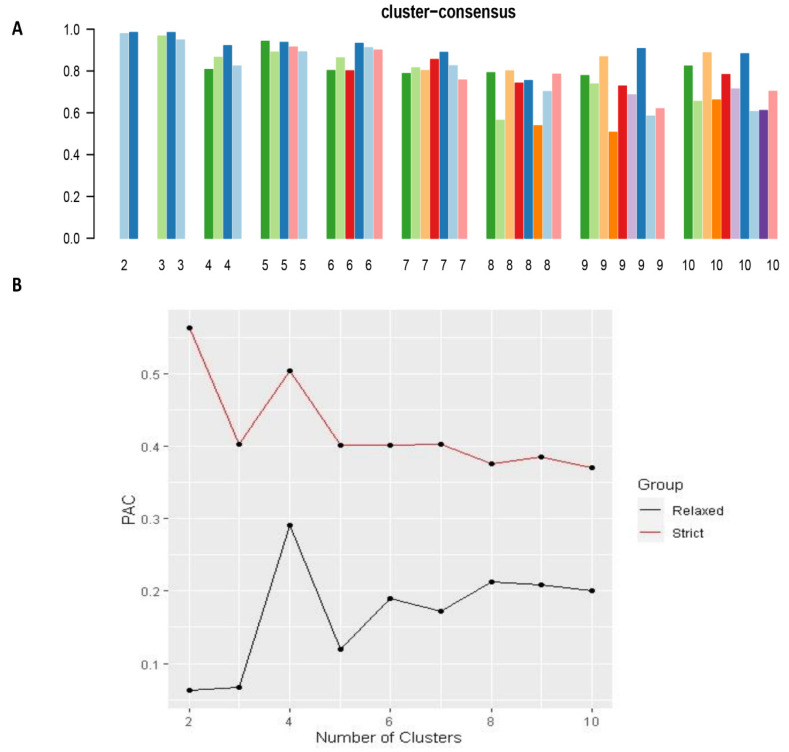
(**A**)The bar plot represents the mean consensus score for different numbers of clusters (k ranges from two to ten); (**B**) The PAC values assess ambiguously clustered pairs.

The CDF plot displays consensus distributions for each k (Figure 1A). The delta area plot shows the relative change in the area under the CDF curve (Figure 1B). The largest changes in the area occurred between k = 3 and k = 5, at which point the relative increase in the area became noticeably smaller. As shown in the CM heatmap (Figure 2, Supplementary Figures S1–S9), the ML algorithm identified cluster 2 and cluster 3 with clear boundaries, indicating good cluster stability over repeated iterations. The mean cluster consensus score was comparable between the scenario of two and three clusters (Figure 3A). Cluster 3 had favorably lower PACs for both relaxed and strict criteria than cluster 2 (Figure 3B), demonstrating that cluster 3 was a less ambiguous cluster compared with cluster 2. Thus, using baseline variables at hospital admission, the consensus clustering analysis identified three clusters that best represented the data pattern of hospitalized patients with hyperchloremia on admission.

**Figure 4:**
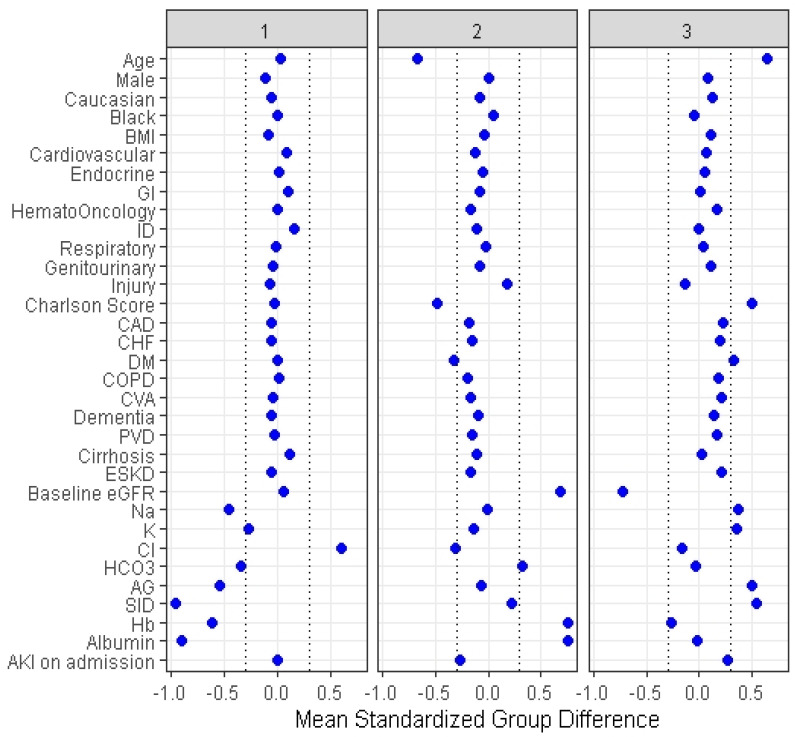
The standardized differences across three clusters for each of the baseline parameters. The *x* axis is the standardized differences value, and the *y* axis shows baseline parameters. The dashed vertical lines represent the standardized differences cutoffs of <−0.3 or >0.3. Abbreviations: AKI, acute kidney injury; DM, diabetes mellitus; COPD, chronic obstructive pulmonary disease; CVA, cerebrovascular accident; PVD, peripheral vascular disease; CHF, congestive heart failure; MI, myocardial infarction; BMI, body mass index; Hb, hemoglobin; SID, strong ion difference; AG, anion gap; ESKD, end stage kidney disease; HCO3, bicarbonate; Cl, chloride; K, potassium; Na, sodium; GFR, glomerular filtration rate; RS, respiratory system; ID, infectious disease; GI, gastrointestinal.

Cluster 1 included 3237 (28%) patients, cluster 2 consisted of 4059 (36%) patients, and cluster 3 had 4098 (36%) patients. Table 1 demonstrates the clinical characteristics of the three identified clusters. Although the distribution of all clinical characteristics significantly differed among the three clusters, some characteristics were considered key features of each cluster with a standardized mean difference of >0.3 (Figure 4). Cluster 1 had higher serum chloride but lower serum sodium, bicarbonate, and anion gap (AG) and strong ion difference (SID), hemoglobin, and albumin. Cluster 2 had younger age, lower comorbidity score, lower serum chloride, and higher estimated glomerular filtration (eGFR), hemoglobin, and albumin. Cluster 3 had older age; higher comorbidity score, particularly diabetes mellitus; higher serum sodium, potassium, AG, SID; and lower eGFR.

**Figure 5:**
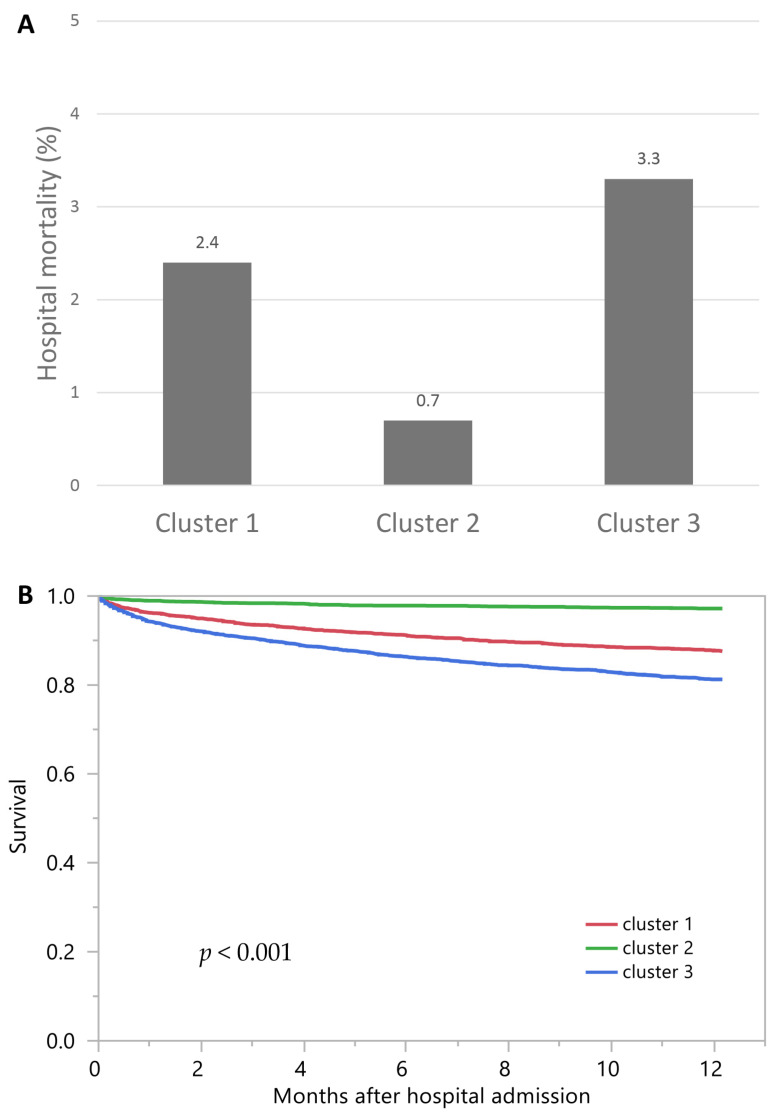
(A) Hospital mortality among different clusters of admission Hyperchloremia; (B) One-year mortality among different clusters of admission hyperchloremia.

Cluster 3 had the highest hospital mortality (3.3%), followed by cluster 1 (2.4%) and cluster 2 (0.7%) (Figure 5A). The ORs for hospital mortality, compared with cluster 2, were 3.60 (95% CI 2.33–5.56) in cluster 1 and 4.83 (95% CI 3.21–7.28) in cluster 3 (Table 2). Similarly, cluster 3 had the highest one-year mortality (18.8%), followed by cluster 1 (12.5%) and cluster 2 (2.8%) (Figure 5B). The HRs for one-year mortality, compared with cluster 2, were 4.49 (95% CI 3.53–5.70) for cluster 1 and 6.96 (95% CI 5.56–8.72) for cluster 3 (Table 2).

## 4. Discussion

Using an unsupervised ML consensus clustering approach, we successfully identified three distinct clusters of hospitalized patients with admission hyperchloremia with high stability and low ambiguity. These three clusters had different baseline characteristics and were associated with different hospital mortality and one-year mortality risks.

Despite the association between hyperchloremia and adverse outcomes [8,9,10], mortality risks among patients with hyperchloremia have not consistently been demonstrated [2,9,13,14,15,16,17,18,19,20,21]. The findings from our study also suggest that patients with hyperchloremia on hospital admission have different characteristics and outcomes. The mortality risk, both in-hospital and one-year mortality, was highest among patients in cluster 3. These patients also had lower baseline eGFR and higher prevalence of AKI on hospital admission. Among all clusters, hyperchloremic patients in cluster 3 had the highest serum sodium and potassium levels. Given that these hyperchloremic patients had more AKI on hospital admission and higher serum sodium with relatively normal bicarbonate (22.5 +/−3.5 mEq/L), it is possible that in cluster 3, hyperchloremia was in the setting of net water losses, including fever, perspiration, inadequate water intake, or (less commonly) diabetes insipidus.

Patients in cluster 1 had the highest serum chloride level among the three clusters. These patients had the lowest serum bicarbonate level with a normal anion gap. In addition, these patients also had the lowest hemoglobin and serum albumin on hospital admission among the three groups. Thirteen percent of these patients had AKI on hospital admission, higher than the prevalence of AKI in cluster 2 but lower than cluster 3. Compared with patients in cluster 2, patients in cluster 1 also carried an increased risk of in-hospital and one-year mortality. Based on patients’ characteristics, they could have received more intravenous 0.9% NaCl for fluid resuscitation before admission, resulting in hyperchloremic metabolic acidosis. While RTA can also cause hyperchloremic (normal anion gap) metabolic acidosis, it is usually a chronic medical condition rather than acute presentation to the hospital [1,10]. In addition, AKI, as occurred in cluster 1, is not a typical presentation of patients with RTA [1,10].

Previous studies presented conflicting findings on mortality risk among patients with hyperchloremia [2,9,13,14,15,16,17,18,19,20,21]. The results of our study also suggest different mortality risks among patients with hyperchloremia. Patients with hyperchloremia in cluster 2 had the lowest in-hospital (0.7%) and one-year mortality (2.8%). These patients were the youngest and had fewer comorbidities and the mildest degree of hyperchloremia. Given that mortality risks are increased among hyperchloremic patients in clusters 1 and 3, future studies are needed on interventional targets to improve outcomes of hyperchloremia among patients with phenotypes in these clusters.

There are several limitations related to this study. First, this was a single-center study, and our patient populations were predominantly white. In addition, given that the data of the hospitalized patients were collected between 2011 and 2013, future studies with more up-to-date datasets are required to confirm our findings. Second, ML clustering was performed at hospital admission, and data before hospitalization (including administration of 0.9% NaCl solution) were limited. Nonetheless, identifying distinct phenotypes in patients with hyperchloremia may provide potential implications for managing and following patients with hyperchloremia, so that such hyperchloremic patients with a high-risk of mortality can be followed up with greater attention, although future studies are required to evaluate the application of this approach in clinical practice.

## 5. Conclusions

ML consensus clustering analysis identified three clusters of hospitalized patients with admission hyperchloremia. These three distinct phenotypic and clinicopathological clusters of patients with admission hyperchloremia are associated with different in-hospital and one-year mortality risks.

## Figures and Tables

**Table 1:** Clinical characteristics according to the distinct clusters of hyperchloremia patients.

Patient Characteristics	Overall (*n* = 11,394)	Cluster 1 (*n* = 3237)	Cluster 2 (*n* = 4059)	Cluster 3 (*n* = 4098)	*p*-Value
Age (years)	60.8 (17.9)	61.3 (15.9)	48.9 (16.5)	72.3 (12.3)	<0.001
Male sex	5706 (50)	1446 (45)	2047 (50)	2213 (54)	<0.001
Race					<0.001
- White	10,528 (92)	2944 (91)	3668 (90)	3916 (96)	
- Black	191 (2)	56 (2)	94 (2)	41 (1)	
- Others	675 (6)	237 (7)	297 (7)	141 (3)	
BMI (kg/m^2^)	29.0 (6.8)	28.4 (6.6)	28.7 (6.7)	29.7 (6.9)	<0.001
Principal diagnosis					<0.001
- Cardiovascular	3750 (33)	1192 (37)	1094 (27)	1464 (36)	
- Endocrine/metabolic	195 (2)	61 (2)	40 (1)	94 (2)	
- Gastrointestinal	974 (9)	367 (11)	253 (6)	354 (9)	
- Genitourinary	403 (4)	93 (3)	84 (2)	226 (6)	
- Hematology/oncology	1233 (11)	348 (11)	234 (6)	651 (16)	
- Infectious disease	379 (3)	198 (6)	54 (1)	127 (3)	
- Respiratory	243 (2)	92 (2)	72 (2)	109 (3)	
- Injury/poisoning	1933 (17)	467 (14)	970 (24)	496 (12)	
- Other	2284 (20)	449 (14)	1258 (31)	577 (14)	
Charlson Comorbidity Score	1.6 (2.2)	1.6 (2.0)	0.5 (1.0)	2.7 (2.6)	<0.001
Comorbidities					
- Coronary artery disease	859 (8)	193 (6)	117 (3)	549 (13)	<0.001
- Congestive heart failure	785 (7)	178 (6)	122 (3)	485 (12)	<0.001
- Peripheral vascular disease	374 (3)	92 (3)	25 (1)	257 (6)	<0.001
- Dementia	169 (1)	29 (1)	13 (0)	127 (3)	<0.001
- Stroke	858 (8)	203 (6)	125 (3)	530 (13)	<0.001
- COPD	807 (7)	244 (8)	85 (2)	478 (12)	<0.001
- Diabetes mellitus	2040 (18)	586 (18)	220 (5)	1234 (30)	<0.001
- Cirrhosis	347 (3)	164 (5)	47 (1)	136 (3)	<0.001
- End-stage kidney disease	378 (3)	77 (2)	11 (0)	290 (7)	<0.001
Laboratory test					
- eGFR (mL/min/1.73 m^2^)	74 (24)	75 (21)	91 (18)	56 (20)	<0.001
- Sodium (mEq/L)	141 (3)	139 (3)	141 (3)	142 (3)	<0.001
- Potassium (mEq/L)	4.2 (0.6)	4.0 (0.6)	4.1 (0.4)	4.4 (0.7)	<0.001
- Chloride (mEq/L)	110 (3)	112 (4)	109 (2)	110 (2)	<0.001
- Bicarbonate (mEq/L)	23 (3)	22 (4)	24 (3)	23 (4)	<0.001
- Anion gap	8 (4)	6 (4)	8 (3)	10 (3)	<0.001
- Strong ion difference	34.8 (3.4)	31.5 (3.0)	35.6 (2.5)	36.7 (2.6)	<0.001
- Hemoglobin (g/dL)	11.5 (2.2)	10.2 (1.9)	13.2 (1.6)	10.9 (1.9)	<0.001
- Albumin (g/dL)	3.5 (0.4)	3.1 (0.3)	3.8 (0.3)	3.5 (0.3)	<0.001
Acute kidney injury	1542 (14)	434 (13)	170 (4)	938 (23)	<0.001

**Table 2:** Mortality outcomes according to the distinct clusters of hyperchloremia patients.

	Hospital Mortality	OR (95% CI)	1-Year Mortality	HR (95% CI)
Cluster 1	2.4%	3.60 (2.33–5.56)	12.5%	4.49 (3.53–5.70)
Cluster 2	0.7%	1 (ref)	2.8%	1 (ref)
Cluster 3	3.3%	4.83 (3.21–7.28)	18.8%	6.96 (5.56–8.72)

## Data Availability

Data are available upon reasonable request to the corresponding author.

## Supplementary Materials

The following are available online at https://www.mdpi.com/article/10.3390/medicina57090903/s1, Figures S1–S9: Consensus matrix heat map depicting consensus values on a white to blue color scale of each cluster.
